# DIANA-mAP: Analyzing miRNA from Raw NGS Data to Quantification

**DOI:** 10.3390/genes12010046

**Published:** 2020-12-30

**Authors:** Athanasios Alexiou, Dimitrios Zisis, Ioannis Kavakiotis, Marios Miliotis, Antonis Koussounadis, Dimitra Karagkouni, Artemis G. Hatzigeorgiou

**Affiliations:** 1 DIANA Lab, Department of Computer Science and Biomedical Informatics, University of Thessaly, 35131 Lamia, Greece; thanosalexiou@uth.gr (A.A.); ikavakiotis@gmail.com (I.K.); mariosmiliwtis@gmail.com (M.M.); dkaragkouni@uth.gr (D.K.); 2 Hellenic Pasteur Institute, 11521 Athens, Greece; dzisis1986@gmail.com; 3 Department of Electrical & Computer Engineering, University of Thessaly, 38221 Volos, Greece; akoussounadis@gmail.com

**Keywords:** bioinformatics, NGS, small RNA-Seq, microRNA, analysis, pipeline, expression, quantification

## Abstract

microRNAs (miRNAs) are small non-coding RNAs (~22 nts) that are considered central post-transcriptional regulators of gene expression and key components in many pathological conditions. Next-Generation Sequencing (NGS) technologies have led to inexpensive, massive data production, revolutionizing every research aspect in the fields of biology and medicine. Particularly, small RNA-Seq (sRNA-Seq) enables small non-coding RNA quantification on a high-throughput scale, providing a closer look into the expression profiles of these crucial regulators within the cell. Here, we present DIANA-microRNA-Analysis-Pipeline (DIANA-mAP), a fully automated computational pipeline that allows the user to perform miRNA NGS data analysis from raw sRNA-Seq libraries to quantification and Differential Expression Analysis in an easy, scalable, efficient, and intuitive way. Emphasis has been given to data pre-processing, an early, critical step in the analysis for the robustness of the final results and conclusions. Through modularity, parallelizability and customization, DIANA-mAP produces high quality expression results, reports and graphs for downstream data mining and statistical analysis. In an extended evaluation, the tool outperforms similar tools providing pre-processing without any adapter knowledge. Closing, DIANA-mAP is a freely available tool. It is available dockerized with no dependency installations or standalone, accompanied by an installation manual through Github.

## 1. Introduction

Emerging technological developments during the last fifteen years, and more specifically, Next-Generation Sequencing (NGS) technologies have led to inexpensive and massive data production, revolutionizing every research aspect in the fields of biology and medicine. Extensive sequencing produced by large consortia [1] has turned microRNAs (miRNAs) into a research hotspot. NGS RNA sequencing has been widely utilized and some protocol pipelines such as the “Tuxedo” pipeline [2] and the “new Tuxedo” package [3] have been the prevalent protocols for expression analysis in RNA-Seq, facilitating a relatively straightforward approach. The plethora of publicly available data has strengthened data-driven research, making appropriate and efficient computational analysis of biological data a strong factor in research. At the same time, researchers trained otherwise may miss this opportunity due to various reasons, ranging from complicated software installation or configuration to a lack of high computing power.

miRNAs are small noncoding RNAs, approximately 22 nts long, considered central post-transcriptional regulators of gene expression [4]. They are abundant in many organisms and are implicated in a variety of physiological and pathological processes. During the past decade, their role has been widely researched in complex diseases, such as cancer, and several studies have reported specific miRNAs to act either as oncogenes or tumor suppressors [5]. Small RNA sequencing (sRNA-Seq) enables the wide-scale quantification of small noncoding RNAs, ~18–30 nucleotide-long RNA molecules [6], providing new insights concerning the function of crucial regulators. Due to miRNAs’ short length, thorough data preprocessing is very important in sRNA-Seq as adapters may affect a significant portion of the reads. Moreover, the high abundance of sequences able to map in multiple places in the genome poses a challenge in the quantification process, one that can lead to significant misinterpretations of data.

Several sRNA-Seq analysis pipelines have been developed throughout recent years. Based on their preprocessing, they can be split into two categories regarding adapter identification. The majority of the programs cover the basic steps of analysis regarding preprocessing, and they are usually specialized in specific analysis parts, such as isomiR detection and handling (sRNAnalyzer [7], QuickMIRSeq [8], Prost! [9], Jasmine [10]), exogenous sequences and different noncoding RNA detection (sRNAnalyzer, sRNAtoolbox [11], mirTools 2.0 [12]), or de novo miRNA identification (CAP-miRSeq [13], miRge 2.0 [14]). Their preprocessing steps, while almost always present, revolve around the basic removal of an adapter assumed to be known, which is not the case when users do not analyze their own data. Some tools such as sRNAnalyzer provide a set of a few widely used adapters in sRNA-Seq studies as choices when the adapter is not known. sRNAnalyzer uses the frequencies of sequences found to infer the primers for multiplexed datasets. MiARma-Seq [15] provides mRNA as well as small RNA analysis with an emphasis on de novo molecule identification. To our knowledge, it is the only tool that currently provides sophisticated adapter-agnostic preprocessing analysis by utilizing Minion, part of the Kraken toolset [16], in order to infer the adapter using sequence frequencies. No additional preprocessing is applied to remove possible remaining adapter contaminants from the data.

In an era where data are produced faster than they are analyzed, more and more scientists are using publicly available datasets for their analyses, leading to a greater need for adapter-agnostic preprocessing analyzing tools. To this end, we developed an automated computational pipeline, DIANA-microRNA-Analysis-Pipeline (DIANA-mAP), which allows the user to perform miRNA NGS data analysis from raw sRNA-Seq data to enable quantification and Differential Expression Analysis (DEA) in an easy, scalable, efficient and intuitive way. It accesses and downloads publicly available datasets from online repositories such as SRA [17], GEO [18] and ENCODE [1] using accession numbers, with an in-house-developed pipeline. The analysis incorporates preprocessing steps, including data quality control, de novo adapter inference and trimming, alignment to the genome of interest and miRNA quantification. Finally, Differential Expression Analysis is performed upon the user’s requisition. DIANA-mAP is free to use under the MIT License and can be acquired through GitHub (https://github.com/athalexiou/DIANA-mAP), with a detailed guide on how to set up and run standalone or, in order to avoid the complicated dependency setups, through a Docker image. In an extended evaluation testing with the miRNA analysis module of miARma-Seq, DIANA-mAP outperforms miARma-Seq in an adapter-agnostic scenario.

## 2. Materials and Methods

In the following sections, we present the utilized resources used to build the DIANA-mAP analysis pipeline and a detailed description of the software.

### 2.1. Utilized Resources

DIANA-mAP provides the option to easily download publicly available data from three widely used online databases, namely Sequence Read Archive (SRA) [17], Gene Expression Omnibus (GEO) [18] and the Encyclopedia of DNA Elements (ENCODE) [1]. SRA is the primary archive that stores raw sequences and alignment results of high-throughput sequencing data. GEO [18] is a publicly available database that stores gene expression datasets. GEO as a public genomics data repository supports many different types of data submissions such as arrays and sequences. ENCODE is an open-access research project aiming to distinguish useful components in human and mouse genomes. The ENCODE project started in 2003 and until now has produced a vast amount of data publicly available through the ENCODE portal. Finally, for the quantification of the analysis, miRBase [19] was used as a reference database of miRNAs. MiRBase is an archive of miRNA sequences and annotations and constitutes the most comprehensive database of its kind, containing more than 15,000 microRNAs and genes, coming from more than 73 different species. Each entry in the miRBase database represents a predicted hairpin of a miRNA transcript, with information on the location and sequence of the mature miRNA.

The pipeline utilizes and incorporates in different steps the following tools: FastQC [20], DNApi [21], Cutadapt [22], Bowtie [23], miRDeep2 [24] and DESeq2 [25]. FastQC provides a standardized and in-depth report of the quality of the reads coming from high-throughput sequencing experiments and aims to produce a straightforward method to perform quality control on them. DNApi is a de novo adapter identification tool that predicts the 3′ adapter sequence. It is based on the notion that the most frequent k-mers are within the adapter sequence of a library. Using these frequencies, it can accurately infer the adapter without any prior metadata knowledge of the library. Additionally, it utilizes statistics such as mapping percentage to strengthen decisions about inferred adapters or indicate the complete absence of adapters in the library. Cutadapt is a publicly available tool designed to trim the adapter sequence on small RNA sequence data. Cutadapt can trim adapters, primers, low-quality bases and other contaminants from a library that can introduce bias to the results of any high-throughput sequencing analysis. Bowtie is a fast and memory-efficient aligner, which aligns large sets of sequence reads to a reference genome. Bowtie returns alignments in SAM or BAM files, and the user can process them for further analysis with tools compatible with this format (SAMtools, BAMtools). miRDeep2 is a software package that identifies canonical and noncanonical miRNAs in an accurate way as well as detects high-confidence candidates in multiple samples. It provides mapping and quantifying capabilities, utilizing Bowtie and miRbase, respectively. Finally, DESeq2 is a method for Differential Expression Analysis of count data, based on the negative binomial distribution, which can be used for quantitative comparisons of interactions between different conditions.

### 2.2. The DIANA-mAP Analysis Pipeline

**Figure 1:**
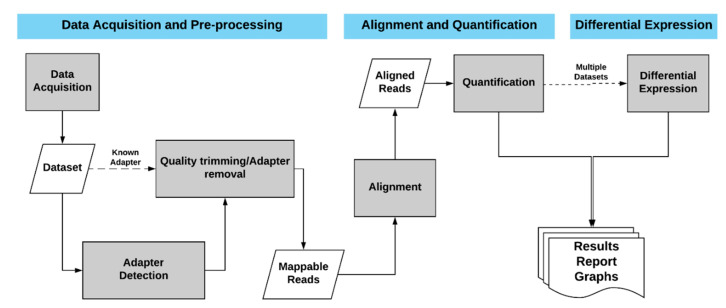
The DIANA-microRNA-Analysis-Pipeline (DIANA-mAP) analysis workflow. The users are able to download or provide their own datasets. If the adapters are not known DIANA-mAP utilizes DNApi to infer them and Cutadapt to remove them. The preprocessed (mappable) reads are aligned to the specified reference genome and then to the known miRNAs from miRBase to provide quantification results. If requested, a Differential Expression (DE) analysis is also performed between the datasets analyzed.

DIANA-mAP is an automated miRNA expression analysis tool that covers the analysis of raw sRNA-Seq data up to quantification. It also offers Differential Expression Analysis on the quantified results if multiple samples under different conditions are introduced. The analysis is performed through three distinct modules, namely (a) *Data Acquisition and Preprocessing*, (b) *Alignment and Quantification*, and (c) *Differential Expression* (Figure 1). The provided results at the end of the analysis, apart from the quantification table and the intermediate results, include graphs and a detailed summary report containing all the important information concerning the analysis. The tool has been developed in R [26], utilizing its rich supporting library sets, and can be run on POSIX systems (Mac, Linux, Unix, BSD) in a single or parallel mode.

#### 2.2.1. Data Acquisition and Preprocessing

The first step is *Data Acquisition* where the user can select from a list of available repositories, i.e., SRA, GEO and ENCODE, to download the desired dataset by providing an accession number. This step is optional if the user does not analyze the in-house-produced data or wishes to extend the spectrum of their analysis by analyzing publicly available resources.

**Figure 2:**
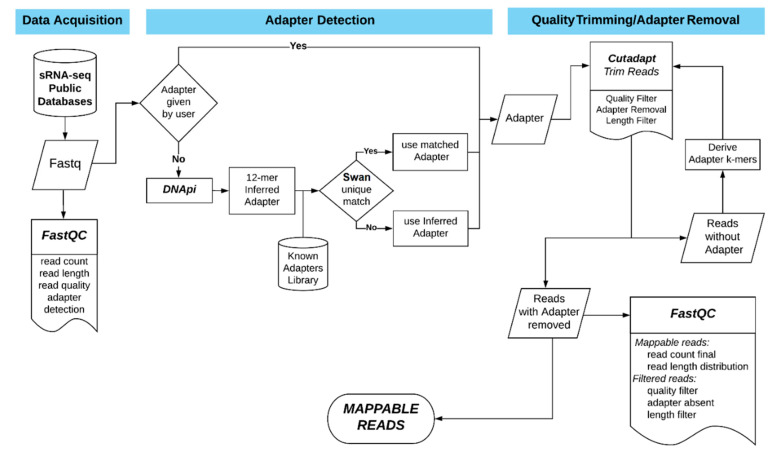
DIANA-mAP preprocessing workflow. It is composed of three individual steps: In the Data Acquisition step, the user can download publicly available datasets from online repositories by providing their accession numbers. The Adapter Detection step either uses a provided adapter sequence or scans the dataset in order to infer the adapter sequence and identify it. The Quality Trimming/Adapter Removal step removes from the dataset low-quality sections and full or partial adapter sequences in order to cleanse the dataset for further analysis.

The next step is the *Quality Check*, performed with the FastQC application [20]. This step provides summary data and graphs depicting the overall quality information of a library. It provides the user with the overall inspection and evaluation of the data before initializing the computationally intensive parts of the analysis. Appropriate warnings are offered to the user, along with the sometimes very useful option to terminate the whole process if the data are of questionable quality (Figure 2).

The third and main step of the first module is *Adapter Detection and Removal*, for which an in-house pipeline was developed. The detection part focuses on identifying the adapter sequences used in the NGS sequencing experiment. If an adapter sequence is provided by the user, either 3′ or 5′, the tool will use that sequence for the removal part. Otherwise, DIANA-mAP utilizes DNApi [21] to infer the 3′ adapter sequence from the dataset using the prevalent sequence frequencies. Using Swan, a short alignment tool from the Kraken software package [16], it cross-references the inferred sequence against a library of known common miRNA adapters, which can always be further enriched by the user. If a known adapter with 90% or higher sequence similarity is found in the library, the tool uses the full known and detected adapter. If not, the 12-mer inferred adapter provided by DNApi is used. Currently, adapter inference through DNApi is available only for 3′ adapters, the most commonly used from major NGS sequencing platforms such as Illumina [27].

Moving on to the adapter removal part of the algorithm, at first, the tool uses Cutadapt [22] in order to trim the low-quality bases of the dataset and remove full instances of the adapter sequence from either the 3′ or 5′ side based on user input. Subsequently, on the remaining reads where no full instance of the adapter is found, k-mers of a provided length (default: 10) are derived from the adapter sequence, and a loop is formed using each of the k-mers as an input to Cutadapt. This process removes fragmented parts of the adapter sequence usually left over from traditional adapter removal approaches, leaving part of the data potentially difficult to assess and use in the following analysis due to lingering contamination. Reads that do not contain a full or a fragmented instance of the adapter are discontinued from the analysis as potential artifacts from the sequencing process. Samples with adapter sequences on both 3′ and 5′ ends, or require other specific treatments for the adapter removal process, represent exceptions that cannot be handled in an automated way and would have to be manually preprocessed beforehand.

Finally, a second round of *Quality Check* is performed using FastQC [20] in order to assess the data status and the progress of the *Adapter Detection and Removal* step by comparing them with the initial *Quality Check* results. This step outputs graphs depicting the progress made and the reads cleansed of adapters, ready to be mapped to the reference genome in the next step of the analysis (mappable reads). The reads cleansed of lingering contamination through the loop described above are subsequently mentioned as “mappable reads (cleansed)” in the produced reports and graphs.

#### 2.2.2. Alignment and Quantification

The next module begins with the *Alignment* step. The cleansed reads provided by the previous module are mapped to the reference genome of the organism in study for verification purposes. This process is accomplished using the mapper script of miRDeep2 [24], which utilizes the Bowtie mapping tool [23] for the alignment.

The final step of DIANA-mAP is *Quantification*, in which miRDeep2 is utilized once more through its quantifier script. For this process, the results of the *Alignment* step are mapped to known mature and precursor miRNAs, acquired from the miRBase database [19], and are transformed into raw and normalized Reads Per Million (RPM), counts per known miRNA as well as log_2_ of RPM.

#### 2.2.3. Differential Expression

In the case of multiple dataset analysis, if requested, an extra module for Differential Expression Analysis can be performed using the *Alignment and Quantification* module results of the analyzed datasets. A condition table is required as input, containing a column with the dataset names along with a column of their respective conditions. DESeq2 [25] software is utilized for this process. Differential Expression Analysis is performed between two different conditions in total and the statistical results are also accompanied by graphs.

#### 2.2.4. Results

**Figure 3:**
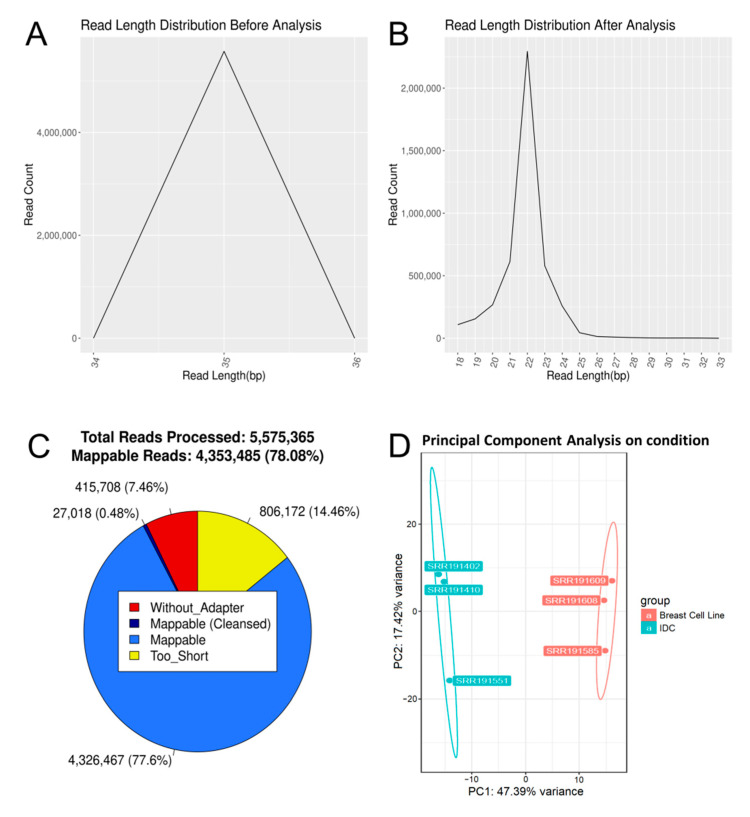
DIANA-mAP visualization results. (**A**) Raw reads length distribution (SRR033716). (**B**) Pre-processed (mappable) reads length distribution (SRR033716). (**C**) Pie-Chart showing the fractions of filtered and mappable reads after the pre-processing step of the analysis (SRR033716). Mappable reads (Cleansed) are reads that were cleansed of partial adapter sequences through pre-processing loops (see Section 2.2.1). “Without_Adapter” are reads in which no adapter was found, while “Too_Short” are reads that had very low number of base pairs (based on configuration) after adapter trimming and were consequently excluded from further analysis. (**D**) Differential Expression Analysis PCA graph for a group of 6 analyzed samples of a miRNA expression study on breast tumors. The three orange-colored samples (SRR191585, SRR191608 and SRR191609) originate from a breast cell line while the three teal-colored ones (SRR191402, SRR191410 and SRR191551) originate from invasive ductal carcinoma (IDC) tissues.

The main results of DIANA-mAP are presented in a table of miRNA expression for each of the provided datasets. Additionally, the user is provided with intermediate statistical results and the subsets of the dataset analyzed are also stored for potential in-depth analysis. Along with a final summarization report, emphasis has been given to the visualization of the results in order to make the analysis easy to follow and understand for beginner and intermediate users alike. The combination of the read length distribution graphs before and after preprocessing provides the user with a quick and comprehensive look at the effects of that analysis step in the data (Figure 3A,B). To further complement that, an overview pie chart of the read distribution after preprocessing is generated (Figure 3C). The Differential Expression Analysis results include overall graphs such as Principal Component Analysis (PCA) between the two conditions as well as expression plots and statistics for the top 100 and top 25 up/downregulated miRNAs. The full detailed list of statistical results for the entirety of the known miRNAs of the organism in study is provided, along with a table of their normalized expression values based on the “median-of-ratios” method [28]. A Differential Expression Analysis example was conducted using 6 samples from a miRNA expression study on breast tumors using deep sequencing [29], the produced PCA plot can be seen in Figure 3D.

## 3. Evaluation

The preprocessing of raw data has long been considered a trivial part of the analysis, and knowledge of the adapters and/or primers used for the sequencing process has been considered as given. Currently, we have reached the era where data are produced faster than they are analyzed, and a significant portion of scientists use publicly available datasets for their studies. As a result, the information about the adapter/primer of a dataset is often missing or incomplete. DIANA-mAP was built with a strong focus on the preprocessing step and provides the possibility to analyze a dataset without any prior information of the adapter used for its production. Mishandlings performed during the preprocessing of a sample directly impact the quantity as well as the quality of the sequencing reads, which are consequently used for the rest of the analysis process. Here, in order to directly measure this, we compare the mapping, quantification and time performance of DIANA-mAP with miARma-Seq [15], currently the only miRNA analysis tool, to our knowledge, that addresses this requirement.

For the evaluation, we used two groups of datasets. The first group, called Dataset_Group_1, contains eight sRNA-Seq libraries (GSE47602) acquired from a study on the human MCF7 cell line that measures the miRNA regulation under hypoxia conditions [30]. Two files of this group are used by miARma-Seq for evaluation, and the other six are provided as examples with the download option of the program. The second group, called Dataset_Group_2, consists of six sRNA-Seq datasets (GSE15229), performed on normal and malignant human B cells [31] and 18 additional datasets acquired through Sequence Read Archive (SRA) and incorporated in functional transcriptomics tool DIANA-miExTra v2.0 [32].

For both programs the analysis was made without providing any adapter information. Also none of the adapters was in the custom library with the known adapters provided by DIANA-mAP (see Section 2.2). Default parameter values were used for both tools with the following exceptions: the trimming quality threshold was set to 20, the minimum and maximum allowed read length were set to 18 and 50 respectively, the maximum allowed mismatch on seed regions during genome alignment was set to 1, the reference genome used was GRCh37 (hg19) and the miRNA reference used was miRBase v20 [19]. The evaluation was made on one core of a High Performance Computer (HPC) with 48 2.3 Ghz-cores and 256 GB of RAM under CentOS operating system.

For the comparison metrics we chose the % of total reads mapped to the genome and the % of total reads that are mapped on known miRNA, also called quantified reads. These statistics characterize the basic results of any analysis tool which also represent a pivotal point for any further analysis such as Differential Expression.

In both groups of datasets we observed 6 (SRR873386, SRR873389 from Dataset_Group_1 and SRR033711, SRR033725, SRR033728, SRR033730 from Dataset_Group_2) with a significant difference of mapped and quantified reads between the two programs (Table 1, Supplementary Table S1). For these 6 datasets, DIANA-map quantified in average 72.5% of total reads compared to an average of only 1.3% for miARma-Seq [15]. Upon closer examination of the results for the aforementioned datasets, a high percentage of reads were mishandled by miARma-Seq during the pre-processing step due to either false inferred adapter and/or too sensitive read cleansing which resulted in too short reads that were subsequently discarded. For the remaining 26 datasets, both tools generate very similar results with the quantified known miRNA raw count numbers showing an insignificant difference of less than 2% (Table 1, Supplementary Table S1).

**Figure 4:**
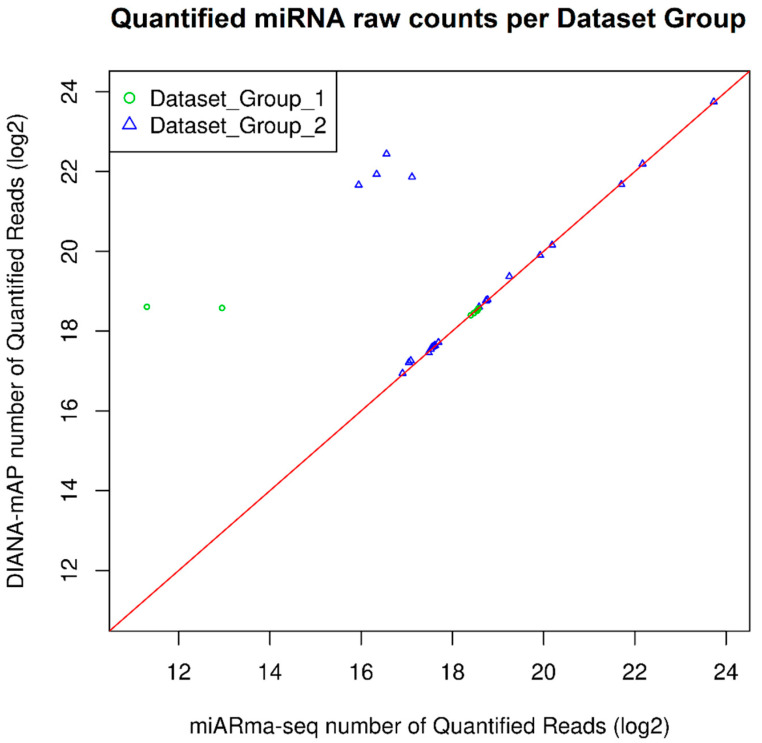
Scatter plot of the quantified miRNA raw counts (Log_2_-transformed) produced by DIANA-mAP and miARma-Seq tools by analyzing: Dataset_Group_1: 8 publicly available datasets analyzed in the publication of miARma-Seq, also offered as example datasets alongside the tool; Dataset_Group_2: 24 publicly available datasets acquired from SRA and analyzed as examples for this study. Each marker represents the number of quantified miRNA raw counts produced by the two tools for a sample. Markers on top of the red line indicate equal numbers of quantified reads between the tools for that sample. Markers skewing toward a particular side indicates a higher number produced for that side.

For 18 out of 32 total samples, information for the adapters used in the original study was provided. Those 18 adapters were correctly predicted by DIANA-mAP, utilizing DNApi’s exhaustive mode, indicating high precision in automatic adapter detection (Supplementary Table S2). For the rest of the 14 samples, the high trimming percentage (>87%), along with the high mapping and quantification percentages in most of them, strongly indicates proper adapter inference from DIANA-mAP (Table 1, Supplementary Tables S1 and S2). In comparison, miARma-Seq shows for 6 out of the 32 samples a significantly lower trimmed percentage statistic along with differences in predicted adapters, providing insight for the difference in performance (Supplementary Table S2). Figure 4 depicts the quantification results for both Groups of Datasets in the form of a scatter plot.

**Figure 5:**
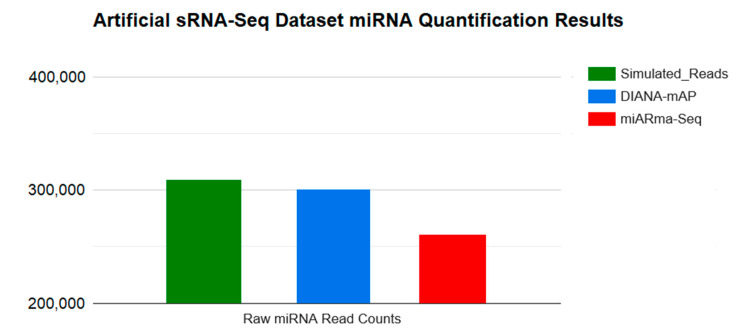
Bar plot of the raw quantified miRNA results for DIANA-mAP and miARma-Seq on an artificial sRNA-Seq dataset. The “Simulated Reads” bar indicates the absolute number of simulated miRNA reads present in the dataset.

Additionally, in order to evaluate the robustness of our tool’s quantified results, we used an artificial sRNA-Seq dataset we created for comparison of the recently published study of the Manatee quantification algorithm [33]. It was generated based on real datasets by employing Monte Carlo random sampling methods. The produced dataset contains 778.072 simulated reads, without adapters, from most of the known small RNA species, including miRNA (39.7%), rRNA (29.3%), tRNA (12.6%) and snoRNA (10.1%). Small random modifications/mismatches have also been introduced to a small fraction of reads. We analyzed the sample using the parameters mentioned above for both DIANA-mAP and miARma-Seq. Despite the high number of nonmiRNA-related simulated reads and read replication numbers, DIANA-mAP was able to automatically identify the absence of adapter sequences using default settings. The quantification results show that DIANA-mAP was able to quantify 38.7% out of the 39.7% miRNAs, included in the artificial dataset, compared to 33.5% for miARma-Seq (Table 2, Figure 5). Moreover, a correlation study of the raw miRNA expression values produced by both tools indicates a higher Pearson correlation of our tool’s results with the simulated read counts compared to miARma-Seq (Table 3).

**Figure 6:**
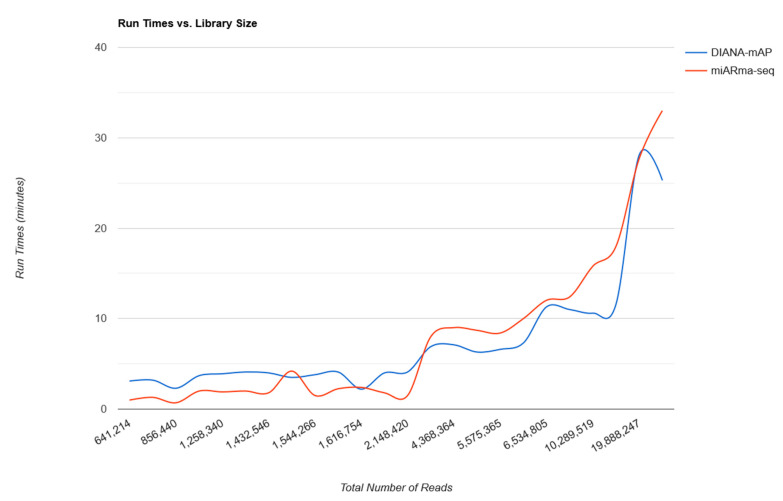
Line graph depicting the analysis run times of the two programs against the datasets’ total number of reads for the 24 libraries in Dataset_Group_2. All the analyses were run using one core of a High-Performance Computer (HPC) with 48 2.3 GHz cores and 256 GB of RAM under the CentOS operating system.

Finally, the evaluation of run time in Dataset_Group_2 for the two programs showed faster performance on deeper sRNA-Seq experiments for DIANA-mAP. On datasets with more than 3 million reads, DIANA-mAP had a run time of 12 min on average compared to 14.8 min for miARma-Seq (Supplementary Table S3). More specifically on the group’s deepest dataset, with 20 million reads (SRR1636963), our tool performed the analysis in 25.3 min versus 33 min for miARma-Seq (Supplementary Table S3). Figure 6 shows the comparative improvement in run times for DIANA-mAP as the datasets increase in size.

## 4. Discussion and Conclusions

Appropriate preprocessing of NGS data is an important prerequisite task for the meaningful analysis of biological and biomedical sequencing data. Errors in this early analysis step will undeniably produce erroneous results and inaccurate conclusions. Here we present DIANA-mAP, a fully automated computational pipeline for sRNA-Seq analysis, with a strong focus on the preprocessing step. It allows miRNA quantification and Differential Expression Analysis to be conducted in an easy, scalable, efficient, and intuitive way. The pipeline has been implemented to support parallelization and is offered dockerized with no dependency installations.

DIANA-mAP performs reliable de novo preprocessing, incorporating extra preprocessing loops to remove adapter contaminants and utilizing a known-adapter-library mechanism that results in more efficient adapter identification even in multiple-adapter dataset group scenarios. It can be used for every organism with a reference genome and microRNA entries in miRBase [19]. Comparison with the widely used, flexible, and multifunctional tool, miARma-Seq [15], showed that DIANA-mAP performed better in an adapter-agnostic scenario. This scenario is expected to grow in occurrence with the day-to-day increase of publicly available datasets and the exponential increase of data-driven studies in all biological and biomedical fields.

## Figures and Tables

**Table 1:** Comparison of raw miRNA read results for DIANA-mAP and miARma-Seq on Dataset Group 1 without adapter information.

Dataset Group 1							
Dataset Accession No.	Number of Reads	Mapped Reads			Quantified Reads		
		miARma-Seq	DIANA-mAP	Difference (% of Total Reads)	miARma-Seq	DIANA-mAP	Difference (% of Total Reads)
SRR873382	500000	410190 (82.04%)	395314 (79.06%)	2.98	384692 (76.94%)	377084 (75.42%)	1.52
SRR873383	500000	404071 (80.81%)	388416 (77.68%)	3.13	379041 (75.81%)	371648 (74.33%)	1.48
SRR873384	500000	376218 (75.24%)	368605 (73.72%)	1.52	363215 (72.64%)	358021 (71.60%)	1.04
SRR873385	500000	365339 (73.07%)	359338 (71.87%)	1.2	346597 (69.32%)	345055 (69.01%)	0.31
SRR873386	500000	10467 (2.09%)	406743 (81.35%)	79.26	7915 (1.58%)	391566 (78.31%)	76.73
SRR873387	500000	416391 (83.28%)	406197 (81.24%)	2.04	389770 (77.95%)	389028 (77.81%)	0.15
SRR873388	500000	408720 (81.74%)	400364 (80.07%)	1.67	389295 (77.86%)	386969 (77.39%)	0.47
SRR873389	500000	3182 (0.64%)	415122 (83.02%)	82.39	2529 (0.51%)	399397 (79.88%)	79.37

Comparison of raw miRNA mapped and quantified read results for DIANA-mAP and miARma-Seq on Dataset Group 1 without adapter information provided. The green-colored difference percentages indicate a higher number of reads for the DIANA-mAP tool compared to the miARma-Seq reads and red-colored ones indicate a lesser number of reads produced.

**Table 2:** Comparison of raw quantified miRNA results for DIANA-mAP and miARma-Seq on an artificial sRNA-Seq dataset.

	Quantified miRNA Reads	% of Total Reads
**Simulated Reads**	308868	39.7
**DIANA-mAP**	300842	38.7
**miARma-Seq**	260821	33.5

Comparison of the raw quantified miRNA results for DIANA-mAP and miARma-Seq on an artificial sRNA-Seq dataset, composed of simulated reads from most of the known small RNA types including miRNA, rRNA, tRNA and snoRNA. Simulated Reads indicate the absolute number of simulated miRNA reads included in the dataset.

**Table 3:** Correlation study of raw miRNA expression results for DIANA-mAP and miARma-Seq on an artificial sRNA-Seq dataset.

	Pearson Correlation Coefficient	Pearson *p*-Value
**DIANA-mAP vs. Simulated Reads**	0.9602398	<2.2 × 10^−16^
**miARma-Seq vs. Simulated Reads**	0.9376623	<2.2 × 10^−16^
**DIANA-mAP vs. miARma-Seq**	0.9231243	<2.2 × 10^−16^

Pearson correlation study of the raw quantified miRNA expression results for DIANA-mAP and miARma-Seq tools on an artificial sRNA-Seq dataset. Simulated Reads indicate the absolute number of simulated miRNA reads, included in the dataset.

## Data Availability

All data for the samples analysed in this study is publicly available through the NCBI Sequence Read Archive (SRA) using the accession numbers provided throughout the manuscript.

## Supplementary Materials

The following are available online at https://www.mdpi.com/2073-4425/12/1/46/s1, Supplementary Table S1: Comparison of raw miRNA read results for DIANA-mAP and miARma-Seq on Dataset Group 2 without adapter information; Supplementary Table S2: Adapter information comparison on the analysis of both dataset group samples by DIANA-mAP and miARma-Seq; Supplementary Table S3: Analysis run-times comparison of both dataset group samples by DIANA-mAP and miARma-Seq.
